# HDL from patients with type 2 diabetes impairs endothelial function by inducing ferroptosis via nuclear receptor coactivator 4

**DOI:** 10.1016/j.jlr.2026.101090

**Published:** 2026-06-25

**Authors:** Zhen-Sheng Ma, Zhi-Wei Mo, Hong-Yu Cao, Yi-Fang Liu, Yue-Ting Kang, Jian-Yu Chen, Chan-Yuan Lyu, Li Chen, Zhi-Jun Ou, Yan Li, Jing-Song Ou

**Affiliations:** 1 Division of Cardiac Surgery, Cardiovascular Diseases Institute, The First Affiliated Hospital, Sun Yat-sen University, Guangzhou, P.R. China; 2 National-Guangdong Joint Engineering Laboratory for Diagnosis and Treatment of Vascular Diseases, NHC key Laboratory of Assisted Circulation and Vascular Diseases (Sun Yat-sen University), Key Laboratory of Assisted Circulation and Vascular Diseases, Chinese Academy of Medical Sciences, Guangdong Provincial Engineering and Technology Center for Diagnosis and Treatment of Vascular Diseases, Guangzhou, P.R. China; 3 Division of Hypertension and Vascular Diseases, Department of Cardiology, Cardiovascular Diseases Institute, The First Affiliated Hospital, Sun Yat-sen University, Guangzhou, P.R. China; 4 Guangdong Provincial Key Laboratory of Brain Function and Disease, Zhongshan School of Medicine, Sun Yat-sen University, Guangzhou, P.R. China

**Keywords:** high-density lipoprotein, ferroptosis, diabetes, endothelial function, mitochondria

## Abstract

Endothelial function is impaired in patients with diabetes. Normal high-density lipoprotein (nHDL) protects endothelial function; however, HDL from patients with diabetes becomes dysfunctional (dHDL) and impairs endothelial function. Ferroptosis is a novel iron-dependent form of cell death driven by the accumulation of lipid peroxidation. Nevertheless, it remains unclear whether dHDL impairs endothelial function by inducing ferroptosis. Human umbilical vein endothelial cells (HUVECs) were treated with nHDL or dHDL; consequently, intracellular levels of ferrous ion, lipid peroxidation, superoxide anions (O_2_^•−^), and nitric oxide (NO) were detected. The expressions of ferritin heavy polypeptide 1 (FTH1), glutathione peroxidase 4 (GPX4), nuclear receptor coactivator 4 (NCOA4), and sphingosine 1-phosphate receptor 1 (S1P1) were measured, while endothelial cell (EC) tube formation was studied. In addition, the mitochondrial membrane potential (MMP), mitophagy, and ferritinophagy were determined. Moreover, vasodilation in C57BL/6 and diabetic db/db mice was examined. dHDL increased ferrous ion levels, lipid peroxidation, O_2_^•−^ production, mitophagy, ferritinophagy, and NCOA4 expression; however, it inhibited FTH1, GPX4, and S1P1 expression, NO generation, and EC tube formation. dHDL decreased MMP and impaired endothelium-dependent vasodilation. These effects of dHDL were reversed by ferrostatin-1 or S1P1 overexpression. The expressions of FTH1, GPX4, and S1P1 decreased, whereas NCOA4 expression increased in diabetic db/db mice compared to wild-type mice. Taken together, dHDL impairs endothelial function by inducing mitochondrial damage and EC ferroptosis through the S1P1–NCOA4 axis. Our findings provide a novel mechanism by which dHDL impairs endothelial function and offer a therapeutic target for diabetic vascular dysfunction.

## Graphical Abstract

HDL from patients with type 2 diabetes mellitus (T2DM) impairs endothelial function by inducing ferroptosis via NCOA4. HDL from patients with T2DM increases the levels of NCOA4 via inhibiting S1P1 to induce ferroptosis, resulting in endothelial dysfunction. It is possible that mitophagy and ferritinophagy may co-regulate ferroptosis during the process.

**Figure:**
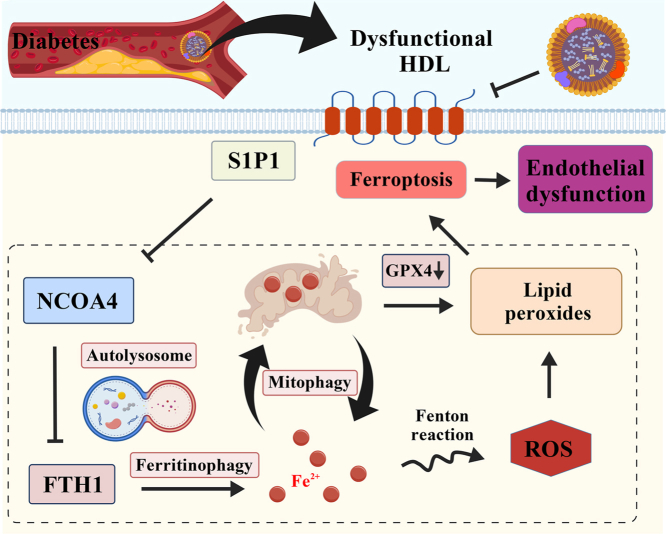

Endothelial dysfunction is a pivotal factor in the initiation and progression of diabetic vasculopathy. In diabetes, multiple factors contribute to insulin resistance and consequent long-term hyperglycemia (1, 2). Oxidative stress and advanced glycation end products (AGEs) are generated during this process, impairing endothelial function and leading to reduced nitric oxide (NO) bioavailability, a pro-inflammatory and pro-thrombotic endothelial phenotype, and reduced angiogenic capacity (3, 4, 5). These pathological changes not only accelerate atherosclerosis and elevate the risks of cardiovascular and cerebrovascular diseases, but also directly cause microvascular damage in diabetic retinopathy, nephropathy, and neuropathy, making the restoration of endothelial function a vital therapeutic objective. Normal high-density lipoprotein (nHDL) can maintain endothelial integrity as a crucial vascular protective factor by promoting NO production, which enhances endothelial proliferation, migration, and tube formation, and exerts antioxidative and anti-apoptotic effects (6, 7, 8, 9, 10). However, HDL undergoes significant compositional and functional changes in diabetes, becoming dysfunctional (dHDL) and losing its ability to protect endothelial function (11, 12, 13). Although this functional loss is well demonstrated, the mechanisms by which dHDL fails to protect endothelial function in patients with diabetes are not fully understood.

Ferroptosis is a unique form of regulated cell death driven by iron-dependent lipid peroxidation (14). Accumulating evidence indicates that ferroptosis plays an important role in the progression of diabetes (15, 16, 17). Long-term diabetes leads to excessive reactive oxygen species (ROS), which in turn results in lipid peroxidation and ferrous ion (Fe^2+^) overloading in tissues and organs (18). Previous studies have confirmed the critical role of ferroptosis in endothelial dysfunction. Our previous study also indicated that PGPC, a pro-inflammatory lipid enriched in atherosclerotic plaques, can induce ferroptosis in ECs to impair endothelial function (19). Therefore, ferroptosis may mediate the impairment of endothelial function induced by dHDL from patients with diabetes.

In the present study, we found that dHDL induced ferroptosis in ECs by downregulating sphingosine 1-phosphate receptor 1 (S1P1) to impair endothelial function. We further found that nuclear receptor coactivator 4 (NCOA4), a critical ferritinophagy regulator, was differently regulated by nHDL and dHDL. dHDL could induce mitochondrial damage and mitophagy by upregulating NCOA4 to accelerate ferroptosis in ECs. Our findings highlight the significance of ferroptosis in HDL-regulated endothelial function, which may serve as a novel potential therapeutic target for patients with diabetes.

## Materials and methods

Please refer to the online supplement for additional details on the methodology.

### Study populations and sample acquisition

Sex- and age-matched patients with type 2 diabetes mellitus (T2DM) with vasculopathy (especially with HbA_1c_ ≥6.5%) and healthy volunteers without diabetes risk factors were recruited for the isolation of HDL. Subjects fasted overnight before blood collection and peripheral venous blood was acquired. The clinical characteristics for subjects are provided in Supplemental Table S1. Informed consent was obtained from all participants in the study. This study was approved by the Ethics Review Board of the First Affiliated Hospital, Sun Yat-sen University. The human studies reported in this study complied with the principles of the Declaration of Helsinki.

### HDL isolation

HDL was isolated from 80 healthy subjects (nHDL) or 80 T2DM patients with diabetes-associated macrovascular complications (dHDL) via sequential ultracentrifugation as described previously (8, 10). HDL was isolated from individual plasma samples. The isolated HDL was stored at −80°C, avoiding repeated freezing and thawing before use. For functional experiments, nHDL and dHDL were randomly selected from the HDL sample pool.

### Cell culture

Human umbilical vein endothelial cells (HUVECs; Cat. DFSC-EC-01, ZQXZBIO) were cultured as described below. All cells were maintained in a humidified atmosphere of 5% CO_2_ and 95% air at 37°C until confluent. Cells were serum-starved for 8 h in ECM containing 0.5% FBS prior to treatment and incubated with 100 μg/ml HDL for 24 h as described previously (19).

### Cell viability assay

The Cell Counting Kit-8 (CCK-8) assay was used to determine whether erastin and dHDL affect cell proliferation. The same number of HUVECs was treated as required in the following experiments. ECM (100 μl) containing 10 μl CCK-8 solution (C0038, China, Beyotime) was added to each well. The absorbance was measured at 450 nm using a microplate reader (Thermo), and comparisons between groups were then made as described previously (19).

### Cell transfection and transduction

Small-interfering RNA (siRNA) targeting *NCOA4* (20 nmol per group) was transfected into HUVECs using Lipofectamine® RNAiMAX Transfection Reagent (Cat. 13778150, Invitrogen) following the manufacturer's instructions. Fresh medium was replaced after transfection for 8 h. Cells were cultured for another 48 h before treatments with the indicated reagents.

Adenoviruses used to overexpress S1P1 were transduced into HUVECs (multiplicity of infection [MOI], 20) for 12 h following the manufacturer's protocol. Cells were cultured for another 72 h before treatments as described previously (7).

### RNA isolation, reverse transcription, and quantitative real-time PCR analysis

RNA was extracted according to the manufacturer's instructions and reverse transcribed into cDNA. Quantitative real-time PCR (qRT-PCR) analysis was performed to quantify the expression levels of various genes (Supplemental Table S2). RT-PCR was performed using a Light-Cycler®480 SYBR Green I Master. All samples were analyzed using a real-time Bio-Rad analyzer as described previously (7).

### Immunoblot analysis

Cells were treated as required, and cellular proteins, including S1P1, NCOA4, FTH1 and GPX4 were detected. Immunoreactive bands were detected using ECL (AI600). Membranes were quantified using ImageJ software (NIH) as described previously (20, 21). In each experimental quantification (e.g., measured grayscale values of protein bands), the results from the experimental groups were compared to those of the control group to obtain ratios. For statistical analysis, the value of the control group within each set was set to 1, and the ratios of the experimental groups relative to the control were used to determine whether the results were statistically significant.

### Measurement of EC nitric oxide (NO) production with 4,5-diaminofluorescein diacetate

HUVECs were cultured on 24-well plates to 90% confluence and then treated as required. We randomly selected six nHDL and six dHDL samples from the HDL sample pool to treat ECs with or without other reagents. Thus, six sets of data were obtained for statistical analysis. When acquiring images for each treatment, including each HDL sample treatment, we selected a field of view that was representative of the average fluorescence intensity of the overall cell population for quantification. This means that only one image per HDL sample treatment was used to obtain the quantitative results for all assays. We used the same method to quantify the image data in all experiments. As a positive control, vascular endothelial growth factor (VEGF; 20 ng/ml; Cat. 293-VE-010, USA, R&D SYSTEMS) was added to the plates for 15 min. 4,5-diaminofluorescein diacetate (DAF-2DA; 10 μM; Merck) was then added to the plates for 30 min. Fluorescence was monitored as a measure of intracellular NO levels using a fluorescence microscope (DMi8; Germany, Leica) and the relative change was analyzed as described previously (10).

### Measurement of EC superoxide anion (O_2_^•−^) production with dihydroethidium

HUVECs were cultured on 24-well plates to 90% confluence and then treated as required. Tumor necrosis factor alpha (TNF-α; 10 μM; Cat. H8916, Sigma-Aldrich) was added as a positive control. Cells were then incubated with dihydroethidium (DHE; 10 μM; Cat. D7008, Sigma-Aldrich) for 30 min at 37°C. Fluorescence was measured, and the relative change was determined as described previously (10).

### EC tube formation assay

EC tube formation was assessed using Matrigel® Matrix (Cat. 354234). HUVECs were digested with trypsin and counted and then were plated at a density of 1–2 × 10^4^/ml in the ECM medium under the indicated stimulation conditions on the Matrigel-coated plates. After incubation for 4–6 h, tube formation was examined by a phase-contrast microscope. Tube formation was quantified using ImageJ software by counting the tube length as described previously (10).

### Detection of mitochondrial membrane potential (MMP)

MMP was measured by the JC-1/MT-1 MitoMP Detection Kit (Cat. MT09/MT13, Japan, DOJINDO). HUVECs were treated as required and stained with JC-1 (2 μM) or MT-1 (0.1%) for 30 min at 37°C protected from light, before image acquisition. Images were obtained through a laser scanning confocal microscope (LSM880, Germany, Carl Zeiss), and the red/green fluorescence intensity ratio was analyzed by ImageJ analysis software as described previously (19).

### Detection of mitophagy

The process of mitophagy was detected using the mitophagy detection kit (Cat. MD01, Japan, DOJINDO). HUVECs were stained with mitophagy dye (100 nM) for 30 min at 37°C protected from light. After cells were treated as required, images were obtained using an LSM880, and the fluorescence intensity ratio was analyzed using ImageJ analysis software.

### Immunofluorescence and confocal microscopy

HUVECs were cultured on confocal dishes and treated as required. The cells were washed with PBS and fixed in 4% paraformaldehyde (Cat. BL539A, China, Biosharp) for 15 min at room temperature. Cells were then permeabilized with 0.2% Triton X-100 (Cat. 9002-93-1, China, Solarbio) for 10 min and blocked with 5% bovine serum albumin (BSA, Cat. AR0009, USA, Bosterbio) for 1 h. Subsequently, cells were incubated with primary antibodies against LC3 and TOM20 overnight at 4°C, followed by incubation with fluorescence-conjugated secondary antibodies for 1 h in the dark. Nuclei were stained with DAPI (Cat. ab104139, abcam) and images were acquired using an LSM980 (22). Colocalization of LC3 and TOM20 was observed and analyzed by confocal fluorescence microscopy.

### Transmission electron microscopy (TEM)

HUVECs were treated as required, and then the cells were collected after centrifugation. The TEM fixative was added to fix the cells at 4°C for 3 h. The cells were embedded in agarose and fixed by 1% OsO_4_ (Cat.18456, USA, Ted Pella Inc) before dehydration at room temperature. The cells were infiltrated and embedded with resin. The resin-containing cells was removed from the embedded mold and were cut into 60–80 nm sections using an ultramicrotome. The sections were stained in 2% uranium acetate and 2.6% lead citrate in order before observation under TEM as described previously (19).

### Measurement of Fe^2+^ content

To measure intracellular Fe^2+^, HUVECs were treated as required and stained with FerroOrange (1 μM; Cat. F374, Japan, DOJINDO) for 30 min at 37°C. The images were obtained using an LSM880, and then were analyzed using the ImageJ software. The mean fluorescence intensity of each group was normalized to that of the control group as described previously (19).

### Lipid peroxides detection

BODIPY® lipid probes (C11-BODIPY; 5 μM; Cat. D3861, Invitrogen) were applied to detect lipid peroxides. HUVECs were treated as required and incubated with C11-BODIPY for 30 min at 37°C protected from light. The reduced BODIPY (R-BODIPY) and the oxidized BODIPY (O-BODIPY) were observed at specific excitation/emission wavelengths as described previously (23). The images were obtained using an LSM980 and then were analyzed using the ImageJ software as described previously (19). The mean fluorescence intensity of each group was normalized to that of the control group.

### Measurement of malondialdehyde (MDA)

An appropriate amount of each sample was treated as required. The MDA detection working solution was added to the sample, mixed, and heated. The sample was then cooled and centrifuged at 1000g for 10 min. The absorbance was measured at 532 nm using a microplate reader. The MDA content of the sample was calculated according to the absorbance of the standard product provided in the lipid peroxidation MDA assay kit (Cat. S0131S, Beyotime).

### Vasodilation study

The experimental protocol was approved by the Animal Ethics Commission of Sun Yat-sen University. Eight-week-old male and female C57BL/6 mice or ten-week-old male BKS-db mice were obtained from GemPharmatech Co., Ltd. For treatment with ferrostatin-1 (Fer-1), the C57BL/6 or BKS-db mice were intraperitoneally injected with 1 mg/kg of Fer-1 every day for 4 weeks (19, 24). Adenovirus particles (2E + 11 v.g.) containing *S1p1* or not (negative control) were designed and synthesized by Shanghai Genechem Co., Ltd. To overexpress S1P1, the C57BL/6 or BKS-db mice received tail-vein injection of 200 μl PBS with adenovirus particles containing *S1p1* or not. After 4 weeks, the aortas were isolated from mice, and then a vasodilation test was performed as described in our previous studies (25, 26). Briefly, four 3-mm-wide aortic rings were obtained and transferred to Krebs-solution. The aortic rings were equilibrated, and the solution was changed. Aortic rings were pretreated under different conditions as indicated in the figure legends. Endothelium-dependent vasodilation was detected with 10^-8^–10^-4^ M acetylcholine (Ach; Cat. A6625, Sigma-Aldrich).

### Statistical analysis

Statistical analyses were performed using GraphPad Prism 8.0 (GraphPad Software). Significant differences (*P* < 0.05) in mean values were determined using Student's *t* test, Welch's *t* test, or Mann-Whitney test for two groups; for more than two groups, one-way analysis of variance (ANOVA) was used and followed by Tukey's test, Welch ANOVA test, or Kruskal–Wallis test. Data are expressed as mean ± SD. In each statistical graph, the specific symbols indicate a statistically significant difference between the groups corresponding to the two ends of the horizontal line on which they are placed.

## Results

### dHDL induces ferroptosis in ECs

**Fig. 1:**
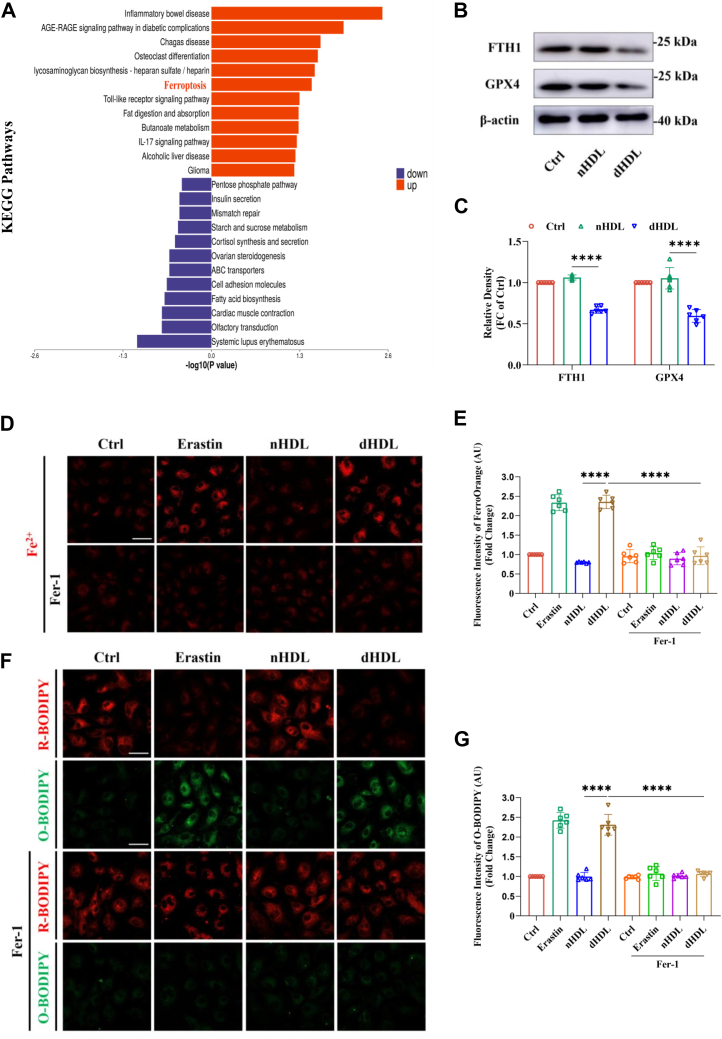
HDL from diabetic patients (dHDL) induces ferroptosis in endothelial cells. A: Human umbilical vein endothelial cells (HUVECs) were treated with HDL (100 μg/ml) from healthy subjects (nHDL) or dHDL for 24 h followed by mass spectrometry and enriched Kyoto Encyclopedia of Genes and Genomes (KEGG) analysis for regulated proteins. B, C: Representative blots and quantification of immunoblot analysis of ferritin heavy polypeptide 1 (FTH1) and glutathione peroxidase 4 (GPX4) in HUVECs treated with nHDL or dHDL for 24 h. D, E: Probe FerroOrange staining fluorescence showed the levels of ferrous ion (Fe^2+^) in cultured HUVECs treated with nHDL or dHDL, with or without ferrostatin-1 (Fer-1, 1 μM) or erastin (5 μM) for 24 h. Representative images and quantification of Fe^2+^ (red) are shown. Erastin was used as a positive control. Fer-1 was used as an inhibitor of ferroptosis. Scale bars, 50 μm. F, G: Probe C11-BODIPY staining fluorescence showed the levels of lipid peroxides in cultured HUVECs treated with nHDL or dHDL, with or without Fer-1 or erastin for 24 h. Representative images and quantification of oxidized BODIPY (O-BODIPY) (green) are shown. Scale bars, 50 μm. Data are presented as the mean ± SD. For (B–G), n = 6. ∗∗∗∗*P* < 0.0001.

A comparative proteomic analysis was performed to evaluate the differentially expressed proteins in HUVECs treated with nHDL or dHDL. The Kyoto Encyclopedia of Genes and Genomes (KEGG) pathway enrichment analysis indicated a significant difference between nHDL- and dHDL-treated cells (Fig. 1A), with ferroptosis emerging as a key pathway implicated in endothelial impairment, consistent with our previous findings (19). Indeed, dHDL downregulated the expression of anti-ferroptosis proteins FTH1 and GPX4 (Fig. 1B, C). Fe^2+^ accumulation and lipid peroxidation are key features of ferroptosis. We used the Fe^2+^-specific fluorescence probe FerroOrange, whose red fluorescence intensity reflects the intracellular Fe^2+^ levels. Lipid peroxidation was evaluated using the probe C11-BODIPY, whose oxidized form (O-BODIPY) emits green fluorescence, with fluorescence intensity proportional to the extent of lipid peroxidation in cells. Erastin, a classic ferroptosis inducer, markedly increased Fe^2+^ levels and lipid peroxidation in HUVECs (Fig. 1D–G). Similarly, dHDL increased intracellular Fe^2+^ levels and oxidative injury in HUVECs (Fig. 1D–G). Ferrostatin-1 (Fer-1) is a classical ferroptosis inhibitor. We found that Fer-1 effectively inhibited both erastin- and dHDL-induced Fe^2+^ accumulation and oxidative injury in HUVECs (Fig. 1D–G). These findings suggest that dHDL can induce ferroptosis in ECs, which is effectively inhibited by Fer-1.

### Suppressing ferroptosis rescues dHDL-induced endothelial dysfunction

**Fig. 2:**
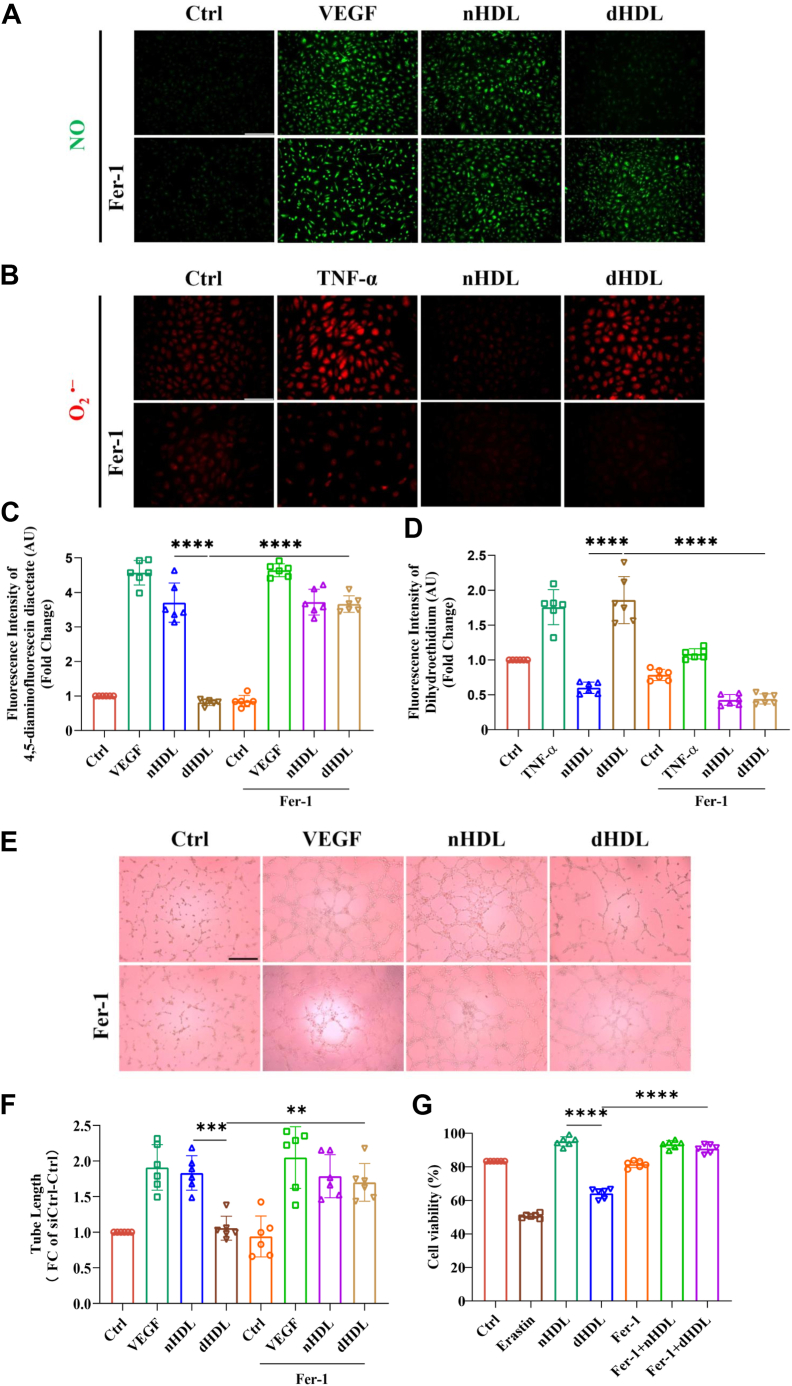
HDL from diabetic patients (dHDL) induces ferroptosis to impair endothelial function. A: 4,5-diaminofluorescein diacetate (DAF-2DA) staining fluorescence showed the levels of nitric oxide (NO) in cultured HUVECs treated with nHDL or dHDL, with or without Fer-1 for 24 h. The representative images of NO (green) are shown. Vascular endothelial growth factor (VEGF, 20 ng/ml) was used as a positive control. Scale bars, 400 μm. B: Dihydroethidium staining fluorescence showed the levels of superoxide anion (O_2_^•−^) in cultured HUVECs treated with nHDL or dHDL, with or without Fer-1 or tumor necrosis factor alpha (TNF-α, 10 μM) for 24 h. The representative images of O_2_^•−^ (red) are shown. TNF-α was used as a positive control. Scale bars, 200 μm. C–D: Quantification of NO and O_2_^•−^ is shown. E: The representative images of tube formation assay in HUVECs treated with nHDL or dHDL, with or without Fer-1 for 24 h. VEGF was used as a positive control. Scale bars, 400 μm. F: Quantification of tube length is shown. G: CCK-8 assays showed viability of HUVECs treated with nHDL or dHDL, with or without Fer-1 or erastin for 24 h. Data are presented as the mean ± SD. For (A–G), n = 6. ∗∗*P* < 0.01; ∗∗∗*P* < 0.001; ∗∗∗∗*P* < 0.0001.

To investigate the role of ferroptosis in HDL-regulated endothelial function, the NO generation, O_2_^•−^ production, tube formation and cell viability were examined. NO is critical for EC proliferation, migration, and vascular integrity maintenance (27). We used DAF-2DA to detect intracellular NO levels, which emits green fluorescence upon reacting with NO. We found that nHDL significantly enhanced NO generation in HUVECs, whereas dHDL was less effective (Fig. 2A, C). Previous studies showed that excess ROS is accumulated in patients with diabetes, which facilitates the Fenton reaction to induce ferroptosis (14, 28). We used DHE to detect intracellular ROS levels, which binds to RNA or DNA after reacting with ROS and emits red fluorescence. As shown in Figure 2B, D, dHDL significantly increased O_2_^•−^ production in HUVECs. Furthermore, nHDL promoted tube formation, whereas dHDL was less effective (Fig. 2E, F), demonstrating that dHDL lost the ability to protect endothelial function. However, Fer-1 restored NO generation, inhibited O_2_^•−^ production, and rescued tube formation in HUVECs in the dHDL treatment group, demonstrating that inhibiting ferroptosis can restore the protective effects of HDL from patients with diabetes on endothelial function. Furthermore, cell viability assays showed that dHDL significantly reduced HUVEC viability, which was rescued by Fer-1 (Fig. 2G). Collectively, these findings suggest that dHDL can induce ferroptosis in ECs to impair endothelial function.

### dHDL increases the expression of NCOA4 to induce EC ferroptosis by downregulating the expression of S1P1

**Fig. 3:**
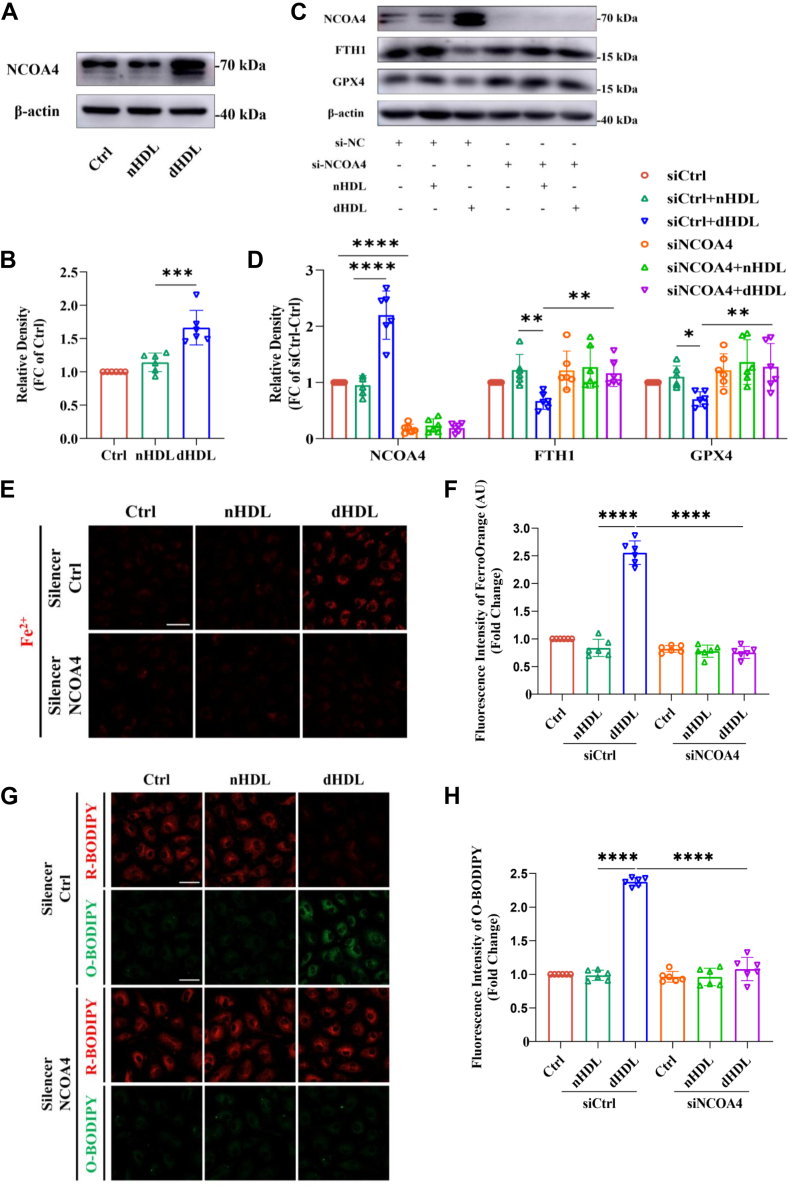
HDL from diabetic patients (dHDL) induces ferroptosis by upregulating the expression of NCOA4 in endothelial cells. A, B: Representative blots and quantification of immunoblot analysis of NCOA4 in cultured HUVECs treated with nHDL or dHDL for 24 h. C, D: Representative blots and quantification of immunoblot analysis of FTH1 and GPX4 in cultured HUVECs treated with nHDL or dHDL for 24 h after silencing NCOA4. E, F: Probe FerroOrange staining fluorescence showed the levels of Fe^2+^ in cultured HUVECs treated with nHDL or dHDL for 24 h after silencing NCOA4. Representative images and quantification of Fe^2+^ (red) are shown. Scale bars, 50 μm. G, H: Probe C11-BODIPY staining fluorescence showed the levels of lipid peroxides in cultured HUVECs treated with nHDL or dHDL for 24 h after silencing NCOA4. Representative images and quantification of O-BODIPY (green) are shown. Scale bars, 50 μm. Data are presented as the mean ± SD. For (A–H), n = 6. ∗*P* < 0.05; ∗∗*P* < 0.01; ∗∗∗*P* < 0.001; ∗∗∗∗*P* < 0.0001.

A previous study demonstrated that NCOA4 plays a pivotal role in ferritinophagy and ferroptosis (29). Therefore, we further investigated the role of NCOA4 in dHDL-induced ferroptosis of ECs. dHDL increased the levels of NCOA4 in HUVECs (Fig. 3A, B). Knocking down NCOA4 significantly attenuated dHDL-reduced FTH1 and GPX4 expression in HUVECs (Fig. 3C, D). Additionally, knocking down NCOA4 reduced dHDL-induced Fe^2+^ accumulation and lipid peroxidation in HUVECs (Fig. 3E–H). Taken together, these data indicated that dHDL induced ferroptosis in ECs by upregulating NCOA4.

**Fig. 4:**
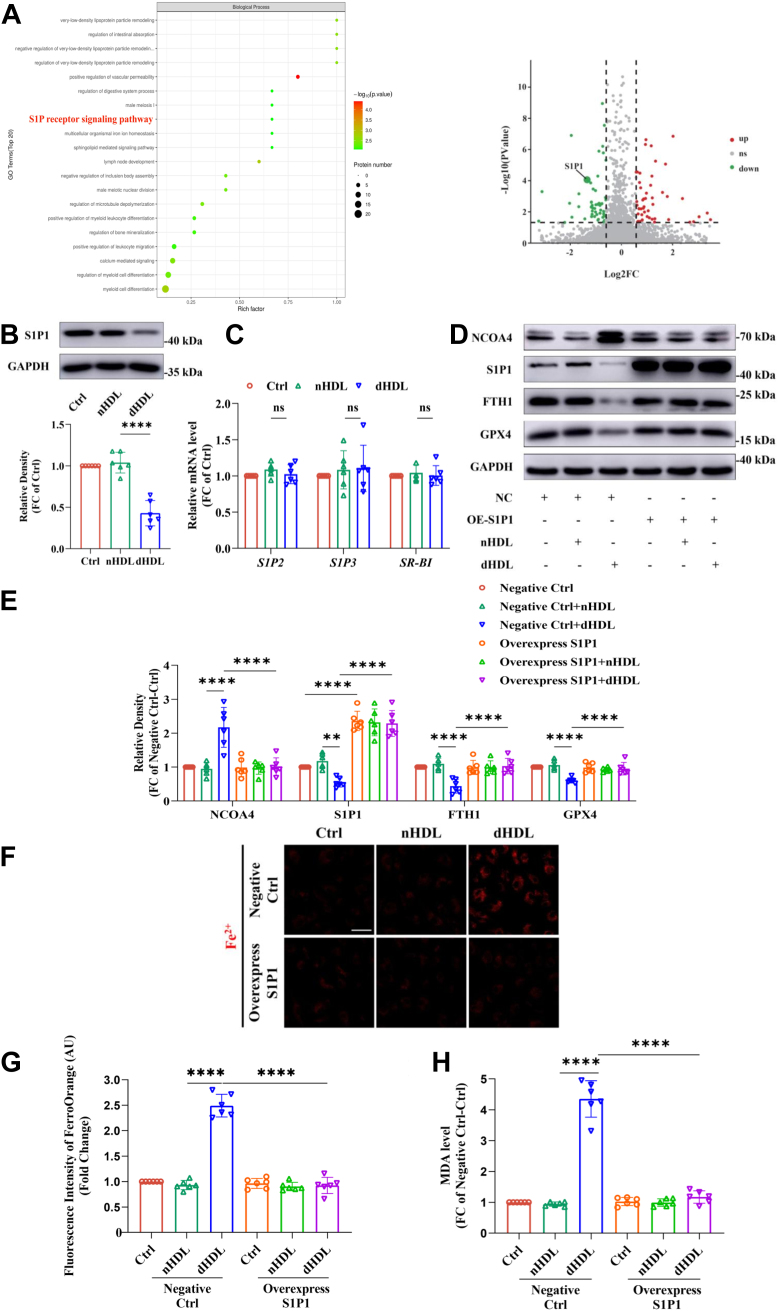
HDL from diabetic patients (dHDL) increases the expression of NCOA4 via S1P1 to induce ferroptosis in endothelial cells. A: HUVECs were treated with nHDL or dHDL for 24 h followed by mass spectrometry, enriched gene ontology (GO) analysis and a Volcano Plot for regulated proteins. B: Representative blots and quantification of immunoblot analysis of S1P1 in HUVECs treated with nHDL or dHDL for 24 h. C: *S1P2*, *S1P3* and *SR-B1* gene expression in HUVECs treated with nHDL or dHDL for 24 h were determined by RT-qPCR. D, E: Representative blots and quantification of immunoblot analysis of NCOA4, FTH1 and GPX4 in HUVECs treated with nHDL or dHDL for 24 h after overexpressing S1P1. F, G: Probe FerroOrange staining fluorescence showed the levels of Fe^2+^ in cultured HUVECs treated with nHDL or dHDL for 24 h after overexpressing S1P1. Representative images and quantification of Fe^2+^ (red) are shown. Scale bars, 50 μm. H: The content of malondialdehyde (MDA) in cultured HUVECs treated with nHDL or dHDL for 24 h after overexpressing S1P1. Data are presented as the mean ± SD. For (B–H), n = 6. ∗∗∗∗*P* < 0.0001; ns, not significant.

Proteomic analysis showed significant enrichment of S1P receptor signaling pathway in gene ontology (GO) enrichment analysis, and S1P1 was downregulated by dHDL in the comparative proteomic analysis (Fig. 4A, B). S1P1 is the most abundant G protein-coupled receptor (GPCR) in ECs and plays a crucial role in maintaining endothelial function (30). S1P4 and S1P5 are mainly expressed in other tissues rather than ECs (31). It has been reported that scavenger receptor class B type 1 (SR-B1), as one of the common HDL receptors, mediates the protective effect of HDL on ECs under high-glucose stimulation (32). Therefore, we investigated the transcriptional levels of common subtypes including *S1P2*, *S1P3* and *SR-B1* (Fig. 4B, C). S1P1 was significantly downregulated in dHDL-treated HUVECs, whereas the transcriptional levels of other receptors did not change in HUVECs treated with nHDL or dHDL. Previous studies showed that HDL-associated S1P and its receptors are critical mediators of endothelial protection in diabetes (33). To determine whether dHDL regulated ferroptosis via S1P1 signaling pathway, we overexpressed S1P1 in HUVECs. Overexpressing S1P1 attenuated dHDL-induced downregulation of FTH1 and GPX4, upregulation of NCOA4 and Fe^2+^ accumulation in HUVECs (Fig. 4D–G). dHDL increased MDA, a well-established biomarker of lipid oxidation, which was attenuated by S1P1 overexpression in HUVECs (34) (Fig. 4H). These findings suggest that dHDL increases the expression of NCOA4 to induce EC ferroptosis by downregulating the expression of S1P1.

**Fig. 5:**
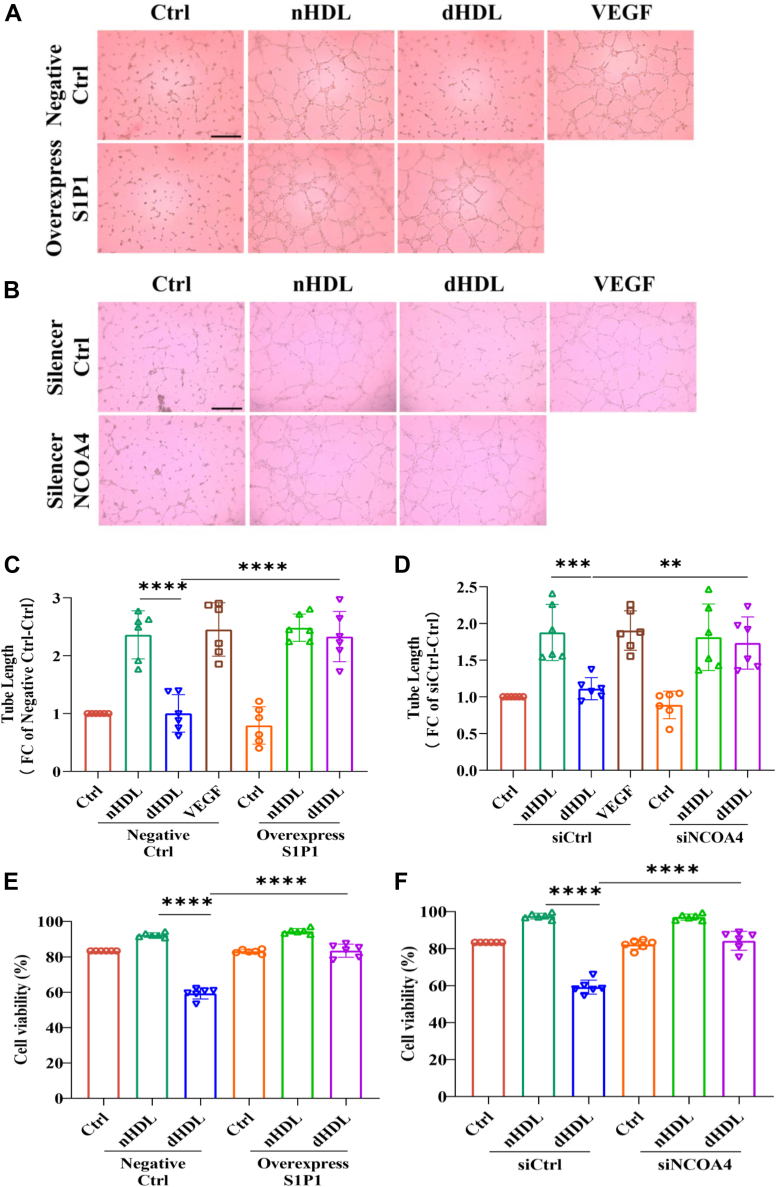
Overexpression of S1P1 or knockdown of NCOA4 attenuates HDL from diabetic patients (dHDL)-impaired endothelial function A: The representative images of tube formation assay in HUVECs treated with nHDL or dHDL for 24 h after overexpressing S1P1. VEGF was used as a positive control. Scale bars, 400 μm. B: The representative images of tube formation assay in HUVECs treated with nHDL or dHDL for 24 h after silencing NCOA4. VEGF was used as a positive control. Scale bars, 400 μm. C, D: Quantification of tube formation assay in HUVECs treated with nHDL or dHDL for 24 h after overexpressing S1P1 or silencing NCOA4. E, F: CCK-8 assays showed viability of HUVECs treated with nHDL or dHDL for 24 h after overexpressing S1P1 or silencing NCOA4. Data are presented as the mean ± SD. For (A–F), n = 6. ∗∗*P* < 0.01; ∗∗∗*P* < 0.001; ∗∗∗∗*P* < 0.0001.

Next, we further investigated the role of S1P1 and NCOA4 in dHDL-regulated endothelial dysfunction. To assess the angiogenic capacity of ECs, we performed tube formation assays and measured the total length of tubular networks formed on Matrigel. Overexpressing S1P1 or knocking down NCOA4 reversed dHDL-inhibited EC tube formation (Fig. 5A–D). In addition, overexpressing S1P1 or knocking down NCOA4 attenuated dHDL-impaired EC viability (Fig. 5E, F). Collectively, these data suggest that S1P1–NCOA4 axis is an important mediator of dHDL-impaired endothelial function.

### dHDL induces mitochondrial damage in ECs

**Fig. 6:**
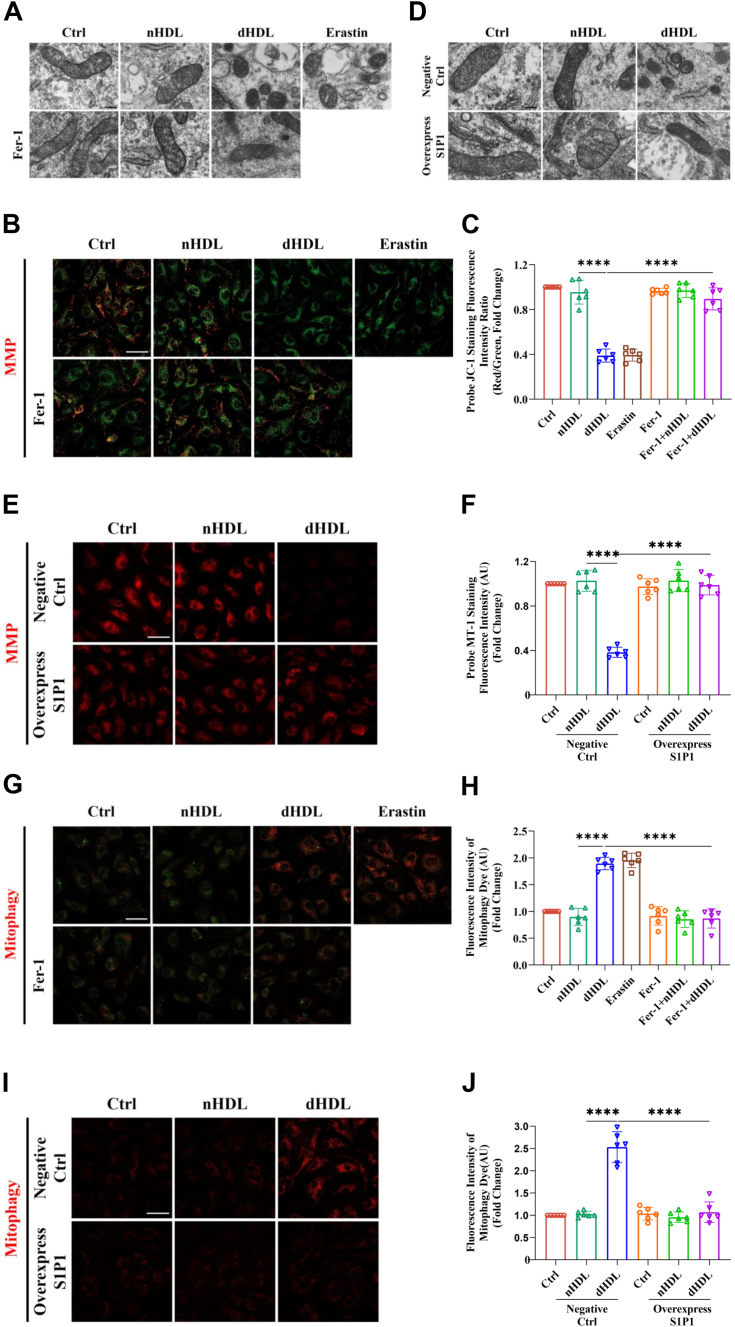
HDL from diabetic patients (dHDL) induces mitochondrial damage in endothelial cells. A: Representative transmission electron microscopy (TEM) images of mitochondria in HUVECs treated with nHDL or dHDL, with or without Fer-1 or erastin for 24 h. Scale bars, 0.2 μm. (n = 5). B, C: Probe JC-1 staining fluorescence showed the levels and quantitation of mitochondrial membrane potential (MMP) in cultured HUVECs treated with nHDL or dHDL, with or without Fer-1 or erastin for 24 h. The representative images of MMP (red) are shown. Scale bars, 50 μm. D: Representative TEM images of mitochondria in HUVECs treated with nHDL or dHDL for 24 h after overexpressing S1P1. Scale bars, 0.2 μm. (n = 5). E, F: Probe MT-1 staining fluorescence showed the levels and quantification of MMP in cultured HUVECs treated with nHDL or dHDL for 24 h after overexpressing S1P1. The representative images of MMP (red) are shown. Scale bars, 50 μm. G, H: The representative images of mitophagy (red) and quantification in HUVECs treated with nHDL or dHDL, with or without Fer-1 or erastin for 24 h. Scale bars, 50 μm. I, J: The representative images of mitophagy (red) and quantification in HUVECs treated with nHDL or dHDL for 24 h after overexpressing S1P1. Scale bars, 50 μm. Data are presented as the mean ± SD. For (B, C, E–J), n = 6. ∗∗∗∗*P* < 0.0001.

Mitochondria play a critical role in the process of ferroptosis. We found that dHDL induced characteristic ferroptosis-associated mitochondrial morphological changes in HUVECs, including mitochondrial shrinkage and increased membrane density via TEM (Fig. 6A) (35). Functionally, mitochondrial energy metabolism is changed, and the ROS production, mitochondrial fusion and fission, mitochondrial membrane potential (MMP), as well as mitophagy are altered during ferroptosis (36). MMP was detected using the JC-1 fluorescent probe in the present study. High MMP leads to probe aggregation to emit red fluorescence, whereas low MMP results in monomeric probe to produce green fluorescence. The red-to-green fluorescence intensity ratio was used to quantify MMP, with a decreased ratio representing impaired mitochondrial function. We found significant MMP dissipation and mitochondrial depolarization in dHDL-treated HUVECs by MMP assays (Fig. 6B, C). These effects were effectively inhibited by overexpressing S1P1 in HUVECs (Fig. 6D–F). Mitophagy is considered a potential activator of ferroptosis (37, 38). We used a mitophagy detection reagent, in which the dye's fluorescence is enhanced in the acidic environment of lysosomes. The red fluorescence intensity can be used to indicate mitophagy activity. dHDL also induced mitophagy, which was inhibited by Fer-1 (Fig. 6G, H). Overexpressing S1P1 attenuated dHDL-induced mitophagy in HUVECs (Fig. 6I, J). These data indicate that dHDL can induce mitochondrial damage and mitophagy to accelerate ferroptosis by inhibiting S1P1 in ECs.

### dHDL induces ferritinophagy and mitophagy to accelerate ferroptosis in ECs

**Fig. 7:**
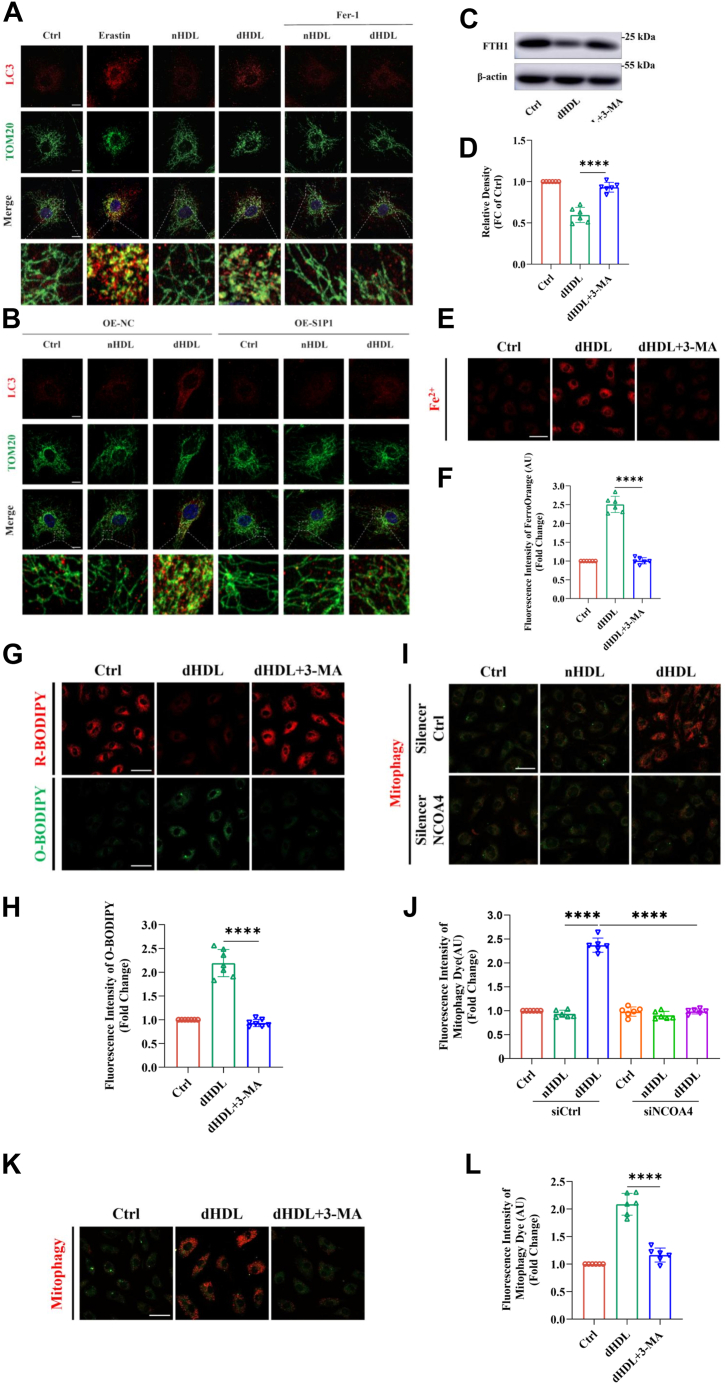
HDL from diabetic patients (dHDL) induces ferritinophagy and mitophagy to accelerate ferroptosis in endothelial cells. A: HUVECs were treated with nHDL, dHDL or erastin, with or without Fer-1 for 24 h. The representative confocal microscopy images of LC3 (red), TOM20 (green), and DAPI (blue) are shown. Magnified views of the boxed areas show LC3-TOM20 colocalization (yellow). Scale bars, 10 μm. B: HUVECs were treated with nHDL or dHDL for 24 h after overexpressing S1P1. The representative confocal microscopy images of LC3 (red), TOM20 (green), and DAPI (blue) are shown. Magnified views of the boxed areas show LC3-TOM20 colocalization (yellow). Scale bars, 10 μm. C, D: Representative blots and quantification of immunoblot analysis of FTH1 in HUVECs treated with dHDL, with or without 3-Methyladenine (3-MA) (5 mM) for 24 h. 3-MA was used as the inhibitor of autophagy. E, F: Probe FerroOrange staining fluorescence showed the levels of Fe^2+^ in cultured HUVECs treated with dHDL, with or without 3-MA for 24 h. Representative images and quantification of Fe^2+^ (red) are shown. Scale bars, 50 μm. G, H: Probe C11-BODIPY staining fluorescence showed the levels of lipid peroxides in cultured HUVECs treated with dHDL, with or without 3-MA for 24 h. Representative images and quantification of O-BODIPY (green) are shown. Scale bars, 50 μm. I, J: The representative images of mitophagy (red) and quantification in HUVECs treated with nHDL or dHDL for 24 h after silencing NCOA4. Scale bars, 50 μm. K, L: The representative images of mitophagy (red) and quantitation in HUVECs treated with dHDL, with or without 3-MA for 24 h. Scale bars, 50 μm. Data are presented as the mean ± SD. For (A-F, I, J), n = 6. For (G, H), n = 7. ∗∗∗∗*P* < 0.0001.

To further validate the role of dHDL in inducing mitophagy, as suggested by the above fluorescence assays, we performed immunofluorescence co-staining of the mitochondrial marker TOM20 and the autophagosome marker LC3. Enhanced mitophagy is indicated by an increase in co-localization of TOM20 and LC3 (Fig. 7A). Consistently, overexpressing S1P1 also suppressed dHDL-induced LC3-TOM20 colocalization in HUVECs (Fig. 7B). Figure 7C, D show that dHDL downregulated the expression of FTH1, which was reversed by the inhibitor of autophagy 3-Methyladenine (3-MA). Furthermore, dHDL increased Fe^2+^ accumulation and oxidative damage in HUVECs, which was inhibited by 3-MA (Fig. 7E–H). dHDL-induced mitophagy was also suppressed by knocking down NCOA4 or 3-MA (Fig. 7I–L). These data indicate that dHDL can induce ferritinophagy and mitophagy to accelerate ferroptosis in ECs by upregulating the expression of NCOA4.

### Inhibition of ferroptosis or overexpression of S1P1 improves endothelium-dependent vasodilation in diabetic mice

**Fig. 8:**
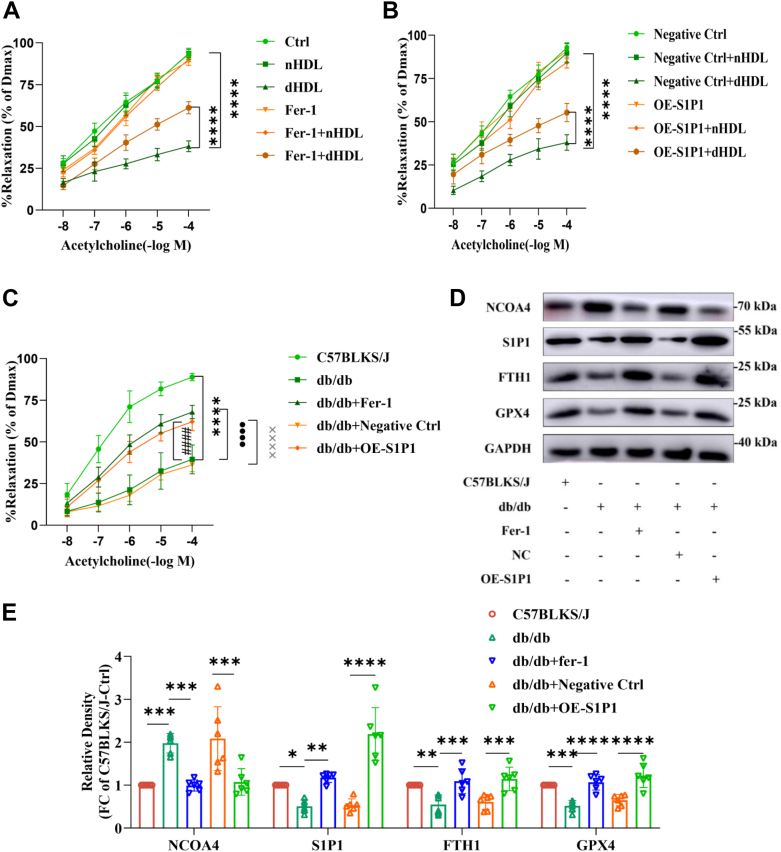
Inhibition of ferroptosis or overexpression of S1P1 improves endothelium-dependent vasodilation in diabetic mice. A: A line chart shows the endothelium-dependent vasodilation of aortic rings ex vivo. Aortic rings isolated from C57BL/6 mice injected with Fer-1 were pretreated with nHDL or dHDL for 30 min, followed by 5-hydroxy tryptamine (5-HT, 5 μM) pre-constriction. Endothelium-dependent vasodilation was detected using acetylcholine (ACh) at different concentrations. n = 8–10, nHDL versus dHDL, dHDL versus dHDL + Fer-1, ∗∗∗∗*P* < 0.0001. B: A line chart shows the endothelium-dependent vasodilation of aortic rings ex vivo. Aortic rings isolated from C57BL/6 mice with S1P1 overexpression were pretreated with nHDL or dHDL for 30 min, followed by 5-HT pre-constriction. Endothelium-dependent vasodilation was detected using ACh at different concentrations. n = 8–10, Negative Ctrl + nHDL versus Negative Ctrl + dHDL, dHDL versus OE-S1P1 + dHDL, ∗∗∗∗*P* < 0.0001. C: A line chart shows the endothelium-dependent vasodilation of aortic rings ex vivo. Aortic rings isolated from diabetic db/db mice overexpressed S1P1 or aortic rings isolated from diabetic db/db mice injected with Fer-1 were pre-constricted by 5-HT. Endothelium-dependent vasodilation was detected using ACh at different concentrations. n = 8–10, C57BLKS/J versus db/db, ∗∗∗∗*P* < 0.0001, db/db versus db/db + Fer-1, ∙∙∙∙*P* < 0.0001, db/db + Negative Ctrl versus db/db + OE-S1P1, ^××××^*P* < 0.0001, db/db versus db/db + OE-S1P1, ^####^*P* < 0.0001. D, E: Representative blots and quantification of immunoblot analysis of S1P1, NCOA4, FTH1 and GPX4 in the aortic tissue of C57BLKS/J, diabetic db/db mice injected with Fer-1 and in the aortic tissue overexpressed S1P1 isolated from diabetic db/db mice. For (A-C, E), n = 6–8. ∗*P* < 0.05; ∗∗*P* < 0.01; ∗∗∗*P* < 0.001; ∗∗∗∗*P* < 0.0001.

We further investigated whether inhibiting ferroptosis could improve endothelium-dependent vasodilation under diabetic conditions. We performed ex vivo aortic ring assays using isolated aortas from C57BL/6 mice or diabetic db/db mice. The aortic rings were first pretreated with HDL or vehicle and then pre-constricted with 5-hydroxy tryptamin (5-HT), followed by acetylcholine (ACh) to induce endothelium-dependent vasodilation. nHDL did not inhibit ACh-induced endothelium-dependent vasodilation, which was largely inhibited by dHDL in C57BL/6 mice. However, this inhibitory effect of dHDL was attenuated in mice treated with Fer-1 or an adenovirus vector to overexpress S1P1 (Fig. 8A, B). Similarly, when diabetic db/db mice were injected with Fer-1 or *S1P1*-overexpressing adenovirus, the ACh-induced endothelium-dependent vasodilation was also improved (Fig. 8C). Furthermore, changes in ferroptosis-related protein levels in aortic tissue of diabetic db/db mice were consistent with those in HUVECs (Fig. 8D, E). The blood glucose and body weight in wild-type mice and diabetic db/db mice are provided in Supplemental Figure S1, which were not changed by Fer-1 or S1P1 overexpression. Collectively, these findings provide evidence supporting the notion that inhibiting ferroptosis or increasing S1P1 expression can partially attenuate the inhibitory effect of dHDL on vascular vasodilation and improve endothelium-dependent vasodilation in diabetic mice.

## Discussion

Endothelial dysfunction is a core event that initiates the inflammatory responses related to vascular complications in diabetic patients (39, 40, 41). In recent years, studies have suggested that ferroptosis plays an important role in atherosclerosis and diabetes (16, 24, 42). EC ferroptosis is a significant factor leading to the impairment of angiogenesis in diabetic wounds (43). Our present study showed for the first time that dHDL induced ferroptosis to impair endothelial function by inhibiting S1P1 expression to upregulate NCOA4, leading to ferritinophagy and mitophagy in diabetes.

HDL not only transports cholesterol but also carries various bioactive proteins, RNAs and lipids (9). Previous studies found that the composition of HDL can change and lose its cardiovascular protection in pathological conditions including atherosclerotic cardiovascular disease, chronic kidney disease, and diabetes (44, 45). Our previous studies demonstrated that HDL from patients with coronary artery disease (CAD) carries miR-24-3p and loses S1P, leading to the loss of its cardiovascular protection (7, 8). Recent studies have suggested that increased miR-181c-5p levels in HDL impaired its protective functions in patients with T2DM (13), and glycosylation modification of apolipoprotein A-IV on HDL induces atherogenesis in patients with T2DM (46). nHDLox (normalized HDL oxidation) was identified recently, whose level positively correlates with the incidence and severity of CAD (47). Our study further found that nHDLox levels positively correlate with fasting blood glucose, suggesting that hyperglycemia can cause functional impairment of HDL (48). These findings suggest that altered HDL composition may lead to its functional impairment in diabetes.

It is well known that HDL interacts with EC receptors including SR-B1 (32) and S1P receptors (S1PRs) to regulate cell activities (7, 49). It has been reported that reconstituted HDL can rescue high glucose-impaired EC tube formation through SR-B1 (32). Another study also showed that nHDL promotes angiogenesis via S1P3 to activate vascular endothelial growth factor receptor 2 (VEGFR2) (49). Our previous studies demonstrated that both miR-24-3p and miR-181a-5p are differentially regulated by nHDL or HDL from patients with CAD via SR-B1 to affect angiogenesis (8, 10). However, we found that dHDL does not affect the expression of SR-B1, but inhibits S1P1 expression to induce ferroptosis, leading to the impairment of endothelial function in the present study. Indeed, we recently also found that HDL-bound S1P can interact with S1P1 to downregulate long non-coding RNA HDRACA expression, thereby promoting angiogenesis, suggesting that dHDL impairs endothelial function via S1P1 (7).

The majority of S1P (65%–80%) is bound to apolipoprotein M on HDL in human plasma (50). Previous studies demonstrated that HDL-bound S1P levels are decreased in patients with T2DM with increasing HbA1c levels (33, 51). Furthermore, another study found that glycation reduced the S1P content of HDL, decreasing the activation of intracellular survival pathways and increasing cardiomyocyte cell death (52). By binding to five known high-affinity receptors, S1P regulates a range of cellular processes including apoptosis, inflammation and vasoprotection (53). As one of these S1P receptors, S1P1 may be a potential therapeutic target for endothelial dysfunction. Indeed, S1P1 directly mediated endothelial activation, leukocyte adhesion molecule expression, and inflammation in acute kidney injury (54), and agonists of S1P1 can improve vascular leakage and protect the integrity of the endothelial barrier (55, 56), suggesting that S1P1 may play a critical role in the protection of endothelial function. In the present study, we also found that dHDL from patients with T2DM inhibited S1P1 expression to impair endothelial function by inducing ferroptosis. However, how dHDL affects the expression of S1P1 and the regulatory role of other HDL receptors on ferroptosis require further study. Therefore, our findings provide a novel potential mechanism by which dHDL induces ferroptosis to impair endothelial function and provide a novel potential therapy for the protection of endothelial function in patients with diabetes.

One of the important findings of the present study is that NCOA4-mediated ferritinophagy serves as a central mechanism for ferroptosis in ECs induced by dHDL from diabetic patients. Ferritin, an iron-storage protein complex in the cytoplasm, is composed of 24 subunits of light (FTL) and heavy (FTH1) chains (57). Ferritin exerts antioxidant effects by storing iron and preventing its engagement in cellular processes. NCOA4 can directly recognize and bind to FTH1, subsequently delivering it to the autophagosome–lysosome pathway for degradation, leading to the accumulation of labile iron ions within the cell (58). A previous study demonstrated that aberrant activation of NCOA4 is a critical regulator of ferroptosis in ECs (29). In the present study, we found that dHDL promoted the expression of NCOA4 by decreasing S1P1 to accelerate ferritinophagy in diabetes. However, the mechanism by which dHDL regulates NCOA4 via S1P1 remains incompletely understood. Notably, it has been reported that activation of JNK can phosphorylate c-Jun and promote its nuclear translocation, thereby upregulating NCOA4 expression (59). Additionally, ataxia telangiectasia mutated protein can enhance NCOA4-ferritin interaction by phosphorylating NCOA4 (60). These regulatory pathways provide important directions for future study to investigate the mechanisms of the dHDL-S1P1-NCOA4 axis.

Mitophagy can selectively transport dysfunctional or superfluous mitochondria to lysosomes for degradation to serve as a cytoprotective mechanism and maintain the balance of intracellular homeostasis (61). Mitophagy can also inhibit the production of lipid peroxidation and then limit ferroptosis by suppressing the accumulation of mitochondrial ROS (62). However, Basit demonstrated that the inhibition of mitochondrial complex I can trigger mitophagy-dependent accumulation of ROS, leading to necroptosis and ferroptosis (63). Mitophagy can also degrade GPX4 to induce ferroptosis (64). Importantly, a previous study demonstrated that de-O-glcNAcylation of the FTH1 at S179 promotes FTH1 binding to NCOA4, thereby accumulating Fe^2+^ for ferroptosis, and mitophagy provides an extra source of labile Fe^2+^ to render the cell more prone to ferroptosis (37). It has been shown that activating S1P1 attenuates kidney ischemia-reperfusion injury by stabilizing mitochondrial function (65). As we found that dHDL can increase NCOA4 through S1P1 to induce ferroptosis, we further investigate the effect of dHDL on mitophagy. We found that dHDL induced mitophagy, and overexpression of S1P1 or silencing NCOA4 reversed dHDL-induced mitophagy. Therefore, it is possible that dHDL induces mitophagy and ferritinophagy to co-regulate ferroptosis in patients with diabetes.

## Conclusion

The present study provides direct evidence that HDL from patients with T2DM increases NCOA4 levels by inhibiting S1P1 to induce ferroptosis, resulting in endothelial dysfunction. Inhibition of ferroptosis or NCOA4 can reverse dHDL-impaired endothelial function in T2DM. Overexpression of S1P1 also suppresses dHDL-induced ferroptosis by inhibiting the expression of NCOA4 in ECs in T2DM. It is possible that dHDL induces mitophagy and ferritinophagy to co-regulate ferroptosis during the process. Our findings reveal a novel mechanism by which HDL from patients with T2DM impairs endothelial function and offer a therapeutic approach for improving endothelial function in patients with T2DM.

## Data availability

The data supporting the findings of this study are presented within the article and its supplementary materials. Additional information or raw data are available from the corresponding author upon reasonable request. Requests for materials should be addressed to MD., PhD. Jing-Song Ou (oujs@mail.sysu.edu.cn), Division of Cardiac Surgery, The First Affiliated Hospital, Sun Yat-sen University, Guangdong, China.

## Supplemental data

This article contains supplemental data (8, 10, 25, 26).

## Conflict of interest

The authors declare that they have no conflicts of interest with the contents of this article.
