# Supplementary material for: HDL from patients with type 2 diabetes impairs endothelial function by inducing ferroptosis via nuclear receptor coactivator 4
Source: J Lipid Res. 2026 Jun 25;67(8):101090. doi: 10.1016/j.jlr.2026.101090 (PMC13396726; doi:10.1016/j.jlr.2026.101090)
Supplement: Supporting Information [file mmc1.docx]

**HDL from patients with type 2 diabetes impairs endothelial function by inducing ferroptosis via nuclear receptor coactivator 4**

**Supplemental Materials and Methods**

**Study populations and sample acquisition**

Sex- and age-matched patients with type 2 diabetes mellitus (T2DM) with vasculopathy (especially with HbA_1c_ ≥6.5%) and healthy volunteers without diabetes risk factors were recruited for the isolation of high-density lipoprotein (HDL). Patients were excluded if they had concomitant infection, advanced liver or kidney failure, other inflammatory or autoimmune disorders, neoplastic disorders, or a history of trauma or major surgery within the previous three months. Subjects fasted overnight before blood collection and peripheral venous blood was acquired. Informed consent was obtained from all participants in the study. Plasma was collected from blood treated with ethylenediaminetetraacetic acid (EDTA) and then stored at -80 °C for further use or subjected to HDL isolation. This study was approved by the Ethics Review Board of the First Affiliated Hospital, Sun Yat-sen University (【2021】190). The human studies reported in this study complied with the Declaration of Helsinki principles.

**HDL isolation**

HDL was isolated from 80 healthy subjects (nHDL) or 80 T2DM patients with diabetes-associated macrovascular complications (dHDL) via sequential ultracentrifugation as described previously (1, 2). HDL was isolated from individual plasma samples. For functional experiments, nHDL and dHDL were randomly chosen from the HDL sample pool. The isolated HDL was stored at −80 °C, avoiding repeated freezing and thawing before use.

**Cell culture**

The human umbilical vein endothelial cells (HUVECs; Cat. DFSC-EC-01, ZQXZBIO, China) were cultured until the fifth passage, then seeded in 96-well, 24-well or 6-well plates and cultured in endothelial cell medium (ECM; Cat. 1001, USA, ScienCell) containing 5% fetal bovine serum (FBS; Cat. 0025, USA, ScienCell), 1% penicillin/streptomycin (P/S; Cat. 0503, USA, ScienCell) and 1% endothelial cell growth supplement (ECGS; Cat. 1052, USA, ScienCell). All cells were maintained in a humidified atmosphere of 5% CO_2_ and 95% air at 37 °C until confluent. Cells were serum-starved for 8 h in ECM containing 0.5% FBS prior to treatment and incubated with 100 μg/mL of HDL for 24 h. Cells were pretreated with 3-Methyladenine (3-MA; 5 mM; Cat. M9281, USA, Sigma-Aldrich) or ferrostatin-1 (Fer-1; 1 μM; Cat. SML0583, USA, Sigma-Aldrich) for 1 h before adding HDL if needed. Cells were treated with erastin (5 μM; Cat. B1524, USA, APExBIO) for 24 h if needed.

**Cell viability assay**

The Cell Counting Kit-8 (CCK-8) assay was used to determine whether erastin and HDL from patients with T2DM (dHDL) affect cell proliferation. The number of HUVECs was determined by cell count plate (Cat. 177-112C, Japan, WATSON). The same number of HUVECs was plated in 96-well plates according to experiments. HUVECs were treated as required in the following experiments. HUVECs were washed twice with PBS, and then 100 μL ECM containing 10 μL CCK-8 solution (C0038, China, Beyotime) was added to each well, and HUVECs were incubated for 4 h at 37 °C. The absorbance was measured at 450 nm using a microplate reader (Thermo), and then comparisons between groups were made. The percentage of living cells in the treated cultures was calculated relative to that in the untreated cultures.

**Cell transfection and transduction**

The small interfering RNA (siRNA) targeting *NCOA4* (20 nmol each group) was designed and synthesized by RiboBio, China, and was transfected into HUVECs using Lipofectamine^®^ RNAiMAX Transfection Reagent (Cat. 13778150, USA, Invitrogen) following the manufacturer's instructions. Cells were seeded into plates at 50-60% confluence, and then were transfected with gene-specific siRNA or control siRNA. The medium was replaced with fresh medium after transfection for 8 h. Cells were cultured for another 48 h before treatments with the indicated reagents. The sequence of the small interfering RNA targeting *NCOA4* used in this study is: GGAAGTGCCTGGTACTGAA.

The adenoviruses used to overexpress S1P1 were purchased from Shanghai Genechem Co., Ltd, and were transduced into HUVECs (multiplicity of infection (MOI) of 20) for 12 h following the manufacturer's protocol. Cells were cultured for another 72 h before treatments.

**RNA isolation, reverse transcription, and quantitative real-time PCR analysis**

RNA was extracted and reverse-transcribed into cDNA. Quantitative real-time PCR (qRT-PCR) analysis was performed to quantify the expression levels of various genes. According to the manufacturer's instructions, total RNA was extracted using the cell RNA rapid extraction kit (Cat. 400-100-100T, China, GOONIE), and the transcriptor first strand cDNA synthesis kit (Switzerland, Roche) was used to synthesize cDNA. RT-PCR was performed using a Light-Cycler®480 SYBR Green I Master (Switzerland, Roche). The primer sequences used are shown in Supplemental Table 2. All samples were analyzed using a real-time Bio-Rad analyzer.

**Immunoblot analysis**

The cells were treated as required, and then washed three times with PBS and lysed by 1 × RIPA lysis buffer (Cat. 9806S, USA, Cell Signaling Technology) supplemented with protease inhibitor (Cat. 539131-1VL, USA, Millipore). Supernatant was collected and protein concentration was measured by bicinchoninic acid protein assay (Cat. 23225, USA, Thermo Fisher Scientific) after centrifugation. Equal amounts of protein mixed with SDS-PAGE loading buffer (Cat. P0015F, China, Beyotime) were loaded and separated by SDS-PAGE. The samples were transferred to PVDF western blotting membranes (Cat. 03010040001, Switzerland, Roche). The membranes were blocked with 5% skimmed milk powder (Cat. S39343-250g, China, ABCone) in Tris-buffered saline with 0.1% Tween^®^20 (TBST) for 1 h at room temperature. The primary antibodies against S1P1 (Cat. ab137467, UK, Abcam), GPX4 (Cat. ab125066, UK, Abcam), FTH1 (Cat. ab65080, UK, Abcam), NCOA4 (Cat. A302-272A, USA, Bethyl), β-Actin (Cat. 4967S, USA, Cell Signaling Technology) and GAPDH (Cat. 60004-1-Ig, USA, Proteintech) were diluted at a ratio of 1:1000 and used for detecting the proteins by incubating membranes overnight at 4 °C. The blots were washed three times with TBST detergent and incubated with a horseradish peroxidase-coupled secondary antibody (HRP Goat anti-rabbit/mouse IgG; 1:10,000; Cat. SA00001-1/SA00001-2, USA, Proteintech) for 1 h at room temperature. Immunoreactive bands were detected using ECL (AI600, GE Healthcare, USA). The membranes were quantified using ImageJ software (NIH, Bethesda, MD, USA). In each experimental quantification (e.g., measured grayscale values of protein bands), the results from the other experimental groups were compared to those of the control group to obtain ratios. For statistical analysis, the value of the control group within each set was set to 1, and the ratios of the experimental groups relative to the control were used to determine whether the results were statistically significant.

**Measurement of endothelial cell (EC)** **nitric oxide (NO) production with 4,5-diaminofluorescein diacetate**

HUVECs were plated on 24-well plates then serum-starved for 8 h in ECM containing 0.5% FBS. Cells were treated with HDL with or without Fer-1 for 24 h. We randomly selected six nHDL and six dHDL samples from the HDL sample pool to treat ECs with or without other reagents. Therefore, six sets of data for statistical analysis. When acquiring images for each treatment, including each HDL sample treatment, we selected a field of view that was representative of the average fluorescence intensity of the overall cell population for quantification. This means that only one image per HDL sample treatment was used to obtain the quantitative results for all assays. We used the same method to quantify image data in all experiments. As a positive control, vascular endothelial growth factor (VEGF; 20 ng/ml; Cat. 293-VE-010, USA, R_&_D SYSTEMS) was added for 15 min. The cells were then incubated with 4,5-diaminofluorescein diacetate (DAF-2DA; 10 µM; USA, Merck) for 30 min at 37 °C following the manufacturer's instructions. Fluorescence was monitored using a fluorescence microscope (DMi8; Germany, Leica) and relative changes were analyzed using ImageJ software.

**Measurement of EC superoxide anion (****O_2_^•-^) production with** **dihydroethidium**

HUVECs were cultured on 24-well plates to 90% confluence, and then serum-starved for 8 h in ECM containing 0.5% FBS. Tumor necrosis factor alpha (TNF-α; 10 µM; Cat. H8916, USA, Sigma-Aldrich) was added as a positive control. Cells were treated with HDL with or without Fer-1 for 24 h. The cells were washed three times with Hank's balanced salt solution (HBSS; Cat. C14175500BT, USA, GIBCO), and then incubated with dihydroethidium (DHE; 10 μM; Cat. D7008, USA, Sigma-Aldrich) for 30 min at 37 °C. Fluorescence was measured using DMi8 and relative changes were determined using the aforementioned ImageJ software.

**EC tube formation assay**

EC tube formation was assessed in 96-well plates using Matrigel^®^ Matrix (Cat. 354234, USA, Corning). Briefly, precooled plates were coated with 60 µL of Matrigel at 4 °C and Matrigel was polymerized at 37 °C for 30 min. Specific genes were knocked down or overexpressed as required in 6-well plates in advance. Cells were digested with trypsin and counted, then plated at a density of 1-2 × 10^4^/mL in the ECM medium with nHDL, dHDL, VEGF (20 ng/ml) or other stimulated conditions on the Matrigel-coated plates. After incubation for 4-6 h, tube formation was examined by a phase-contrast microscope. Tube formation was quantified using ImageJ software by counting the tube length.

**Detection of** **mitochondrial membrane potential (MMP)**

MMP was measured by the JC-1/MT-1 MitoMP Detection Kit (Cat. MT09/MT13, Japan, DOJINDO). Cells were treated as required and stained with JC-1 (2 μM) for 30 min at 37 °C protected from light. The cells were then washed twice with HBSS and incubated in the imaging buffer during image acquisition. For MT-1 staining, confluent cells were stained with MT-1 (0.1%) for 30 min at 37 °C, protected from light, washed twice with HBSS, treated as required, washed twice again with HBSS, and incubated in imaging buffer during image acquisition. Images were obtained through a laser scanning confocal microscope (LSM880, Germany, Carl Zeiss), and the red/green fluorescence intensity ratio was analyzed by ImageJ analysis software. JC-1/MT-1 aggregates form polymers in the mitochondrial matrix of healthy mitochondria and emit intense red fluorescence. However, when mitochondrial membrane potential decreases or is lost in unhealthy mitochondria, JC-1 remains as monomers in the cytoplasm and generates green fluorescence. Changes in the red/green fluorescence ratio reflect differences in mitochondrial membrane potential.

**Detection of mitophagy**

Mitophagy was detected using the mitophagy detection kit (Cat. MD01, Japan, DOJINDO). After confluence, the cells were washed twice with ECM without FBS, and then stained with mitophagy dye (100 nM) for 30 min at 37 °C protected from light. The cells were washed twice with ECM without FBS again. After treating cells as required, images were obtained using a LSM880, and the fluorescence intensity ratio was analyzed using ImageJ analysis software. Normally, mitophagy dye chemically binding to the mitochondria fluoresces weakly. When mitophagy occurs, the impaired mitochondria fuse with lysosomes, and the mitophagy dye produces a strong fluorescence as the PH decreases. Therefore, changes in fluorescence intensity reflect the process of mitophagy.

**Immunofluorescence and confocal microscopy**

HUVECs were cultured on confocal dishes and treated as required. After treatment, cells were washed with PBS and fixed in 4% paraformaldehyde (Cat. BL539A, China, Biosharp) for 15 min at room temperature. Cells were then permeabilized with 0.2% Triton X-100 (Cat. 9002-93-1, China, Solarbio) for 10 min and blocked with 5% bovine serum albumin (BSA) (Cat. AR0009, USA, Bosterbio) for 1 h. Subsequently, cells were incubated with primary antibodies against LC3 (1:500 dilution, Cat. 66139-3-Ig, China, Proteintech) and TOM20 (1:500 dilution, Cat. 11802-1-AP, China, Proteintech) overnight at 4 °C, followed by incubation with fluorescence-conjugated secondary antibodies for 1 h in the dark. Nuclei were stained with DAPI (Cat. ab104139, UK, abcam) (24). Images were acquired using an LSM980. Colocalization of LC3 and TOM20 was observed and analyzed by confocal fluorescence microscopy.

**Transmission electron microscopy (TEM)**

HUVECs were treated as required, and then the cells were collected into a 15-mL EP tube for centrifugation at 1000 rpm for 5 min. After centrifugation, the supernatant was removed, and TEM fixative was added to the cells for fixation at 4 °C for 3 h. The 1% agarose solution was prepared by heating and dissolving in advance. The agarose was then cooled before being added to the EP tube. The fixed cells were suspended with forceps and wrapped in the agarose. The cells were then fixed with 1% OsO_4_ (Cat. 18456, USA, Ted Pella Inc) in PBS for 2 h at room temperature protected from light. The OsO_4_ was then removed, and the cells were rinsed in PBS three times for 15 min each. The cells were dehydrated sequentially with 30%, 50%, 70%, 80%, 95%, 100%, and 100% ethanol at room temperature for 20 min each. And then the cells were treated with 100% acetone (Cat. 10000418, China, SINOPHARM) twice for 15 min each. The cells were infiltrated and embedded in resin. First, a 1:1 mixed solution of acetone and resin (EMBed 812, Cat. 90529-77-4, SPI) was prepared to infiltrate the cells for 2-4 h at 37 °C. Subsequently, a 1:2 mixed solution of acetone and resin was prepared to infiltrate the cells for 12 h at 37 °C. Finally, pure resin was used to penetrate the cells for 5-8 h at 37 °C. After infiltration was completed, pure resin was placed into the embedding mold and the cells were polymerized in a 37 °C oven overnight. The embedding mold was then moved to a 60 °C oven for polymerization for 48 h. The resin containing cells was removed from the embedded mold and was cut into 60-80-nm-thick sections on the ultramicrotome. The sections were placed on the surface of deionized water. The sections were collected onto copper mesh. The copper mesh was then stained for 8 min in 2% uranium acetate (Cat. 02624-AB, SPI) saturated alcohol solution protected from light. Subsequently, the copper mesh was rinsed in 70% ethanol three times, followed by rinsing in ultrapure water for 3 times. The copper mesh was stained in 2.6% lead citrate for 8 min while avoiding CO_2_, followed by rinsing with ultrapure water for 3 times. After being dried with filter paper, the copper mesh was placed in a dryer overnight at room temperature. The copper mesh was observed under the TEM.

**Measurement of Fe**²⁺ **content**

To measure intracellular Fe²⁺, FerroOrange (Cat. F374, Japan, DOJINDO) was used according to the manufacturer's protocol. HUVECs were treated as required and washed three times with HBSS, and then stained with FerroOrange (1 μM) for 30 min at 37 °C. Images were obtained using a LSM880. Relative changes were analyzed using ImageJ software. The mean fluorescence intensity of each group was normalized to that of the control group.

**Detection of lipid peroxides**

BODIPY^®^ lipid probe (C11-BODIPY; 5 μM; Cat. D3861, USA, Invitrogen) was used to detect lipid peroxides. The HUVECs were cultured to 90% confluence and serum-starved for 8 h in ECM containing 0.5% FBS prior to treatment. Then cells were treated with HDL with or without Fer-1 for 24 h or other reagents as required. Then the cells were washed twice with HBSS and incubated with C11-BODIPY for 30 min at 37 °C protected from light. The stained cells were washed twice with HBSS and incubated with ECM without FBS. Reduced BODIPY (R-BODIPY) was observed at excitation/emission wavelengths of 581/591 nm, whereas oxidized BODIPY (O-BODIPY) was observed at excitation/emission wavelengths of 488/510 nm. Images were obtained using a LSM980. Relative changes were analyzed using ImageJ software. The mean fluorescence intensity of each group was normalized to that of the control group.

**Measurement of** **malondialdehyde (MDA)**

Briefly, after HUVECs were pretreated with HDL, the cells were washed twice with PBS and lysed by 1 × RIPA lysis buffer supplemented with protease inhibitor. Supernatant was collected and protein concentration was measured by bicinchoninic acid protein assay after centrifugation. TBA storage solution with a concentration of 0.37% and MDA detection working solution were prepared in advance. An appropriate amount of sample was collected, and a double volume of MDA detection working solution was added. The sample was then mixed and heated in boiling water for 15 min. The sample was cooled to room temperature in a water bath, and centrifuged at 1000 g for 10 min. Two hundred microliters of supernatant was added to the 96-well plate. The absorbance was measured at 532 nm using a microplate reader. The MDA content of the sample was calculated according to the absorbance of the standard provided in the lipid peroxidation MDA assay kit (Cat. S0131S, China, Beyotime). Differences in MDA content among samples were compared based on the MDA content per unit mass of protein.

**Vasodilation study**

The experimental protocol was approved by the Animal Ethics Commission of Sun Yat-sen University. Eight-week-old male and female C57BL/6 mice or ten-week-old male BKS-db mice were obtained from GemPharmatech Co., Ltd. For treatment with ferrostatin-1 (Fer-1), the C57BL/6 or BKS-db mice were intraperitoneally injected with 1 mg/kg of Fer-1 every day for 4 weeks. Adenovirus particles (2E + 11 v. g.) containing *S1P1* or not (negative control) were designed and synthesized by Shanghai Genechem Co., Ltd. To overexpress S1P1, the C57BL/6 or BKS-db mice received tail-vein injections of 200 µL PBS with adenovirus particles containing *S1P1* or not. After 4 weeks, the mice were anesthetized with pentobarbital and euthanized, and the aortas were isolated. Briefly, four 3 mm wide aortic rings were obtained and transferred to Krebs solution (pH 7.4, 119 mM NaCl; 25 mM NaHCO_3_; 1.6 mM CaCl_2_; 4.7 mM KCl; 1.2 mM KH_2_PO_4_; 1.2 mM MgSO_4_⋅7H_2_O; and 11.1 mM D-glucose). The aortic rings were equilibrated for 1 h, and the solution was changed every 15 min. Aortic rings were pretreated with nHDL (100 µg/mL) or dHDL (100 µg/mL) for 30 min as indicated in Figure 8A-B (Figure 8C has no HDL pretreatment). As a control, the aortic rings were not pretreated with HDL. Subsequently, the aortic rings were pre-constricted with 5 µM 5-hydroxy tryptamine (5-HT; Cat. 50679, USA, Sigma-Aldrich). Endothelium-dependent vasodilation was detected with 10^−8^–10^−4^ M acetylcholine (ACh; Cat. A6625, USA, Sigma-Aldrich) as previously described (3, 4).

**Statistical analysis**

Statistical analyses were performed using GraphPad Prism 8.0 (GraphPad Software). Significant differences (*p* < 0.05) in mean values were determined using Student's *t*-test, Welch's *t*-test, or Mann-Whitney test for two groups; for more than two groups, one-way analysis of variance (ANOVA) was used and followed by Tukey's test, Welch ANOVA test, or Kruskal-Wallis test. Data are expressed as mean ± SD. In each statistical graph, the specific symbols indicate a statistically significant difference between the groups corresponding to the two ends of the horizontal line on which they are placed.

**References**

1. Kang B.A., Li H.M., Chen Y.T., Deng M.J., Li Y., Peng Y.M., et al. High-density lipoprotein regulates angiogenesis by affecting autophagy via miRNA-181a-5p. Sci China Life Sci. 2024;67:286-300.

2. Li H.M., Mo Z.W., Peng Y.M., Li Y., Dai W.P., Yuan H.Y., et al. Angiogenic and antiangiogenic mechanisms of high density lipoprotein from healthy subjects and coronary artery diseases patients. Redox Biol. 2020;36:101642.

3. Fu L., Hu X.X., Lin Z.B., Chang F.J., Ou Z.J., Wang Z.P., et al. Circulating microparticles from patients with valvular heart disease and cardiac surgery inhibit endothelium-dependent vasodilation. J Thorac Cardiovasc Surg. 2015;150:666-672.

4. Gao J.J., Wu F.Y., Liu Y.J., Li L., Lin Y.J., Kang Y.T., et al. Increase of PCSK9 expression in diabetes promotes VEGFR2 ubiquitination to inhibit endothelial function and skin wound healing. Sci China Life Sci. 2024;67:2635-2649.

**Supplemental Table S1. Clinical characteristics for isolated HDL**

| Variables | Healthy subjects | T2DM | *p* |
| --- | --- | --- | --- |
| Participants | 80 | 80 |  |
| Male/Female | 48/32 | 59/21 |  |
| MAP (mmHg) | 88.54 ± 2.14 | 87.33 ± 2.2 | NS |
| Fasting glucose (mmol/L) | 4.89 ± 0.14 | 7.98 ± 0.99 | <0.0001 |
| HbA1c (%) | 5.26 ± 0.13 | 7.86 ± 0.36 | <0.0001 |
| Total Cholesterol (mmol/L) | 3.87 ± 0.17 | 3.76 ± 0.18 | NS |
| TG (mmol/L) | 1.19 ± 0.09 | 1.3 ± 0.09 | NS |
| HDL (mmol/L) | 1.19 ± 0.07 | 0.99 ± 0.05 | <0.0001 |
| LDL (mmol/L) | 2.34 ± 0.12 | 2.28 ± 0.14 | NS |
| Creatinine (μmol/L) | 75.23 ± 4.26 | 78.6 ± 3.82 | NS |
| Urea (mmol/L) | 5.52 ± 0.3 | 6.1 ± 0.35 | NS |

MAP: mean arterial pressure; TG: triglyceride; HDL: high density lipoprotein; LDL: low-density lipoprotein.

**Supplemental Table S2. Primers used in the real-time RT-PCR**

| Name | Target | Forward primer | Reverse primer |
| --- | --- | --- | --- |
| *SR-B1* | Human | ACTTCTGGCATTCCGATCAGT | ACGAAGCGATAGGTGGGGAT |
| *S1P2* | Human | GCAAGGCGCAACTTGAGAAA | AAATGTCCCTAGCCACTGCC |
| *S1P3* | Human | AGCCCAAACAAAAACGCTGT | TCCAAAATCCACGAGAGGGC |
| *GAPDH* | Human | AATGGGCAGCCGTTAGGAAA | GCGCCCAATACGACCAAATC |

**Supplemental Figure S1. Blood glucose and body weight in wild-type mice and diabetic db/db mice**

A, B: The bar charts show the blood glucose (A) and body weight (B) levels in C57BLKS/J, diabetic db/db mice, diabetic db/db mice injected with Fer-1, diabetic db/db mice injected with adenovirus particles overexpressing *S1P1*. Blood glucose and body weight were increased in diabetic db/db mice. Data are presented as the mean ± SD. For (A, B), n=6. *****p*<0.0001
